# Comprehensive analysis of m^6^A-regulatory genes in soybean uncovers GmMTBa as a critical determinant of salinity stress tolerance

**DOI:** 10.1016/j.abiote.2026.100046

**Published:** 2026-03-28

**Authors:** Leili Wang, Chengyang Song, Zhu Yan, Tianqi Wang, Yisheng Fang, Xiulin Liu, Junlong Bao, Dan Zhu, Xiao Luo

**Affiliations:** a Shandong Key Laboratory of Precision Molecular Crop Design and Breeding, Peking University Institute of Advanced Agricultural Sciences, Shandong Laboratory of Advanced Agricultural Sciences in Weifang, Weifang, 261325, China; b Shandong Provincial Key Laboratory of Plant Stress, College of Life Sciences, Shandong Normal University, Jinan, 250358, China; c College of Life Science, Key Laboratory of Plant Biotechnology in Universities of Shandong Province, Qingdao Agricultural University, Qingdao, 266109, China; d School of Advanced Agricultural Sciences, Peking University, Beijing, 100871, China

**Keywords:** m^6^A, Writer complex, *GmMTB*, Salinity, Soybean

## Abstract

Soybean (*Glycine max*) is an important protein and oil crop whose yield is significantly affected by salinity stress. N^6^-methyladenine (m^6^A), a prominent epigenetic modification of RNA, exerts crucial regulatory functions in plant development and stress responses. Through genome-wide analysis, we identified 55 m^6^A modification–regulatory genes in soybean across 19 soybean chromosomes, categorized into writers, readers, and erasers. The promoters of these genes are enriched in light-, phytohormone-, and stress-responsive *cis*-elements and display diurnal and tissue-specific expression patterns, suggesting multifaceted regulation. Chromatin immunoprecipitation revealed distinct histone modification signatures associated with these genes, including H3K4me3, H3K36me3, and H2A.Z. Protein–protein interaction analysis confirmed the assembly of core GmMTA–GmMTB–GmFIP37 writer components, with GmFIP37 forming homodimers and heterodimers. Evolutionary analysis showed that *GmMTBa* underwent strong selection pressure with genetic conservation, while *GmMTBb* experienced selection during domestication, showing an association with flowering and yield traits. Notably, we discovered that GmMTBa, a core subunit of the m^6^A methyltransferase complex, functions as a critical determinant of salinity stress tolerance in soybean, as *Gmmtba* mutants exhibited pronounced salinity sensitivity. Mechanistically, GmMTBa regulates the m^6^A modification of *GmMSH1* transcripts, a key suppressor of stress signal transduction. *GmMSH1* knockdown lines were more tolerant to salinity stress, under which conditions *GmMSH1* expression was significantly elevated in *Gmmtba* mutants. These results indicate that GmMTBa influences salinity stress tolerance by modulating *MSH1*-dependent stress signaling pathways. Our findings reveal an m^6^A-mediated regulatory mechanism in plant stress acclimation and establish GmMTBa as a promising candidate for improving soybean resilience to salinity stress.

## 1 Introduction

In the processes that translate genetic information into cellular functions, RNA acts as a crucial intermediary. In addition, RNA is subject to chemical modifications that significantly influence the regulation of gene expression, the study of which is called epitranscriptomics [1]. More than 200 chemical modifications of RNA have been documented, affecting various physiological and biochemical regulatory pathways [1]. Among these modifications, N^6^-methyladenine (m^6^A) is the most prevalent in eukaryotes, spanning mammals, plants, insects, and yeasts. m^6^A profoundly affects RNA metabolism by modulating mRNA stability, splicing, translation, and nucleocytoplasmic partitioning. This dynamic and reversible post-transcriptional modification, akin to DNA methylation, is regulated by three key components: writers, erasers, and readers. Writers are methyltransferases that catalyze the addition of m^6^A to RNA molecules; in Arabidopsis (*Arabidopsis thaliana*), these enzymes include mRNA ADENOSINE METHYLASE (MTA), METHYLTRANSFERASE B (MTB), FKBP12-INTERACTING PROTEIN of 37 kD (FIP37), and FIONA1 (FIO1) [2]. Erasers are demethyltransferases, such as ALKBH9B and ALKBH10B in *Arabidopsis* [3,4], which selectively remove these methylation marks. Readers, including proteins with YT521-B homology (YTH) domains like EVOLUTIONARILY CONSERVED C-TERMINAL REGION 2 (ECT2), ECT3, ECT4, and POLYADENYLATION SPECIFICITY FACTOR 30-L (CPSF30-L), specifically recognize and bind to m^6^A-modified RNA [[5], [6], [7]]. These components, many of which are highly similar to their mammalian counterparts, are crucial to the conserved functions of m^6^A-regulated genes in plants.

The m^6^A modification of RNA regulates many aspects of plant growth and development across diverse species. In *Arabidopsis*, m^6^A modification influences key developmental stages, including embryogenesis [8], flowering time [2,6], proliferation of the shoot stem apex meristem [9], leaf growth and trichome development [10], and root development [11]. Beyond *Arabidopsis*, the m^6^A modification has been implicated in fruit ripening in strawberries (*Fragaria vesca*) via the abscisic acid (ABA) pathway [12], fruit expansion in tomato (*Solanum lycopersicum*) [13], and reproductive development in rice (*Oryza sativa*) [14]. Notably, studies have shown that manipulating m^6^A levels using demethylases has the potential for enhancing crop yields in rice and potato (*Solanum tuberosum*) [15,16]. m^6^A writers, have emerged as critical regulators of plant development. For instance, MTAs modulate reproduction across multiple plant species, influencing seed development, male reproductive organ formation, and fruit ripening [17]. In soybean, for example, GmMTA regulates plant height, mediating shade avoidance responses, with the potential to increase crop yield [18]. These m^6^A components orchestrate plant development by regulating the RNA metabolism of key genes. Specific examples in *Arabidopsis* include the role of FIP37 in stabilizing the transcripts of meristem-related genes like *WUSCHEL* (*WUS*) and *SHOOT MERISTEMLESS* (*STM*) [9] and the control of poly(A) site selection by CPSF30L during floral transition [19]. Moreover, the m^6^A modification contributes to plant stress tolerance, including responses to salinity stress, heat stress, and drought conditions [4,20].

Another crucial epigenetic mechanism that affects cellular functions is histone methylation. Primarily targeting lysine and arginine residues, the consequences of this modification depend on the specific residue, methylation pattern (monomethylation to trimethylation), and genomic context. Histone 3 (H3) is a primary methylation target, with modifications categorized as either activating (H3K4, H3K36) or repressing (H3K9, H3K27) transcription.

The effects of m^6^A modification and histone methylation interact, with this crosstalk varying among species. In mammals, the positions of m^6^A peaks in mRNA correspond to the positions of H3K36me3 modifications associated with the corresponding coding region, with METHYLTRANSFERASE-LIKE 14 (METTL14) directly recruiting RNA polymerase II for co-transcriptional m^6^A deposition [21]. Conversely, in *Arabidopsis*, H3K36me2 peaks in a genomic region correlate with the deposition of m^6^A at the 3' end of newly synthesized transcripts from that region. Furthermore, the H3K36me2 writer component SET DOMAIN GROUP 8 (SDG8) and the m^6^A writer component FIP37 have been reported to physically interact, providing direct evidence for crosstalk between histone methylation and RNA methylation [22]. However, prior studies have primarily focused on how these two epigenetic layers synergistically regulate the expression of target genes. Whether the genes that regulate m^6^A modification are themselves subject to histone modifications and, if so, how these modifications might influence the expression of these regulators remains largely unexplored.

Soybean, a crucial oilseed crop, provides essential nutrients and contributes to sustainable agriculture through nitrogen fixation. RNA m^6^A levels in soybean change dynamically in response to pathogen infection and environmental stress [23]. Previously, we conducted a combined global quantitative proteomics and m^6^A RNA sequencing analysis, which confirmed that the m^6^A modification acts to brake or fine-tune protein synthesis, precisely regulating protein accumulation [24]. Several m^6^A writer components have been identified in soybean [25], but additional m^6^A writers, along with erasers and readers remain to be clearly characterized in this crop. Identifying all the genes responsible for m^6^A RNA modification in soybean is crucial for understanding the regulatory network controlling epigenetic modifications and revealing potential targets for soybean breeding.

A crucial player in epigenetic regulation is MutS Homolog 1 (MSH1), a plant-specific nuclear-encoded protein derived from the bacterial MutS DNA repair system. In *Arabidopsis*, suppression of *MSH1* expression alters epigenetic reprogramming in the nucleus, manifesting through distinct alterations in nuclear DNA methylation patterns, accumulation of small interfering RNAs (siRNAs), and significant changes in the transcriptome [26]. These epigenetic changes affect the expression of genes associated with multiple critical pathways, including circadian rhythms and phytohormone signal transduction [26]. Notably, when *msh1* mutants are used as rootstocks for grafts with wild-type scions, the progeny of the grafted plants exhibit enhanced growth phenotypes, suggesting that loss of MSH1 function coordinates the balance between plant growth and defense [27]. Enhanced growth phenotypes induced by suppression of *MSH1* expression have been observed in diverse plant species, including sorghum (*Sorghum bicolor*) and tomato [26]. RNA interference (RNAi)-mediated suppression of soybean *MSH1* (*GmMSH1*) expression significantly enhances seed yield [28], demonstrating the practical agricultural potential of *MSH1* manipulation in crop improvement. However, the mechanisms controlling *MSH1* expression and whether it is regulated by m^6^A modification remain poorly understood.

In this study, we identified 55 m^6^A regulatory genes in the soybean genome, classifying them as writers, readers, or erasers. We analyzed the expression patterns of m^6^A-related genes and their potential histone modifications. Protein–protein interaction analysis confirmed the assembly of a core GmMTA–GmMTB–GmFIP37 writer complex. We further demonstrate that GmMTBa, a core methyltransferase subunit, confers tolerance to salinity stress by regulating the m^6^A modification of *GmMSH1* transcripts. Our findings provide critical insights into the epigenetic and post-transcriptional regulatory mechanisms underlying soybean acclimation and stress resilience.

## 2 Results

### 2.1 Genome-wide identification and evolutionary analysis of m^6^A regulatory genes

**Fig. 1:**
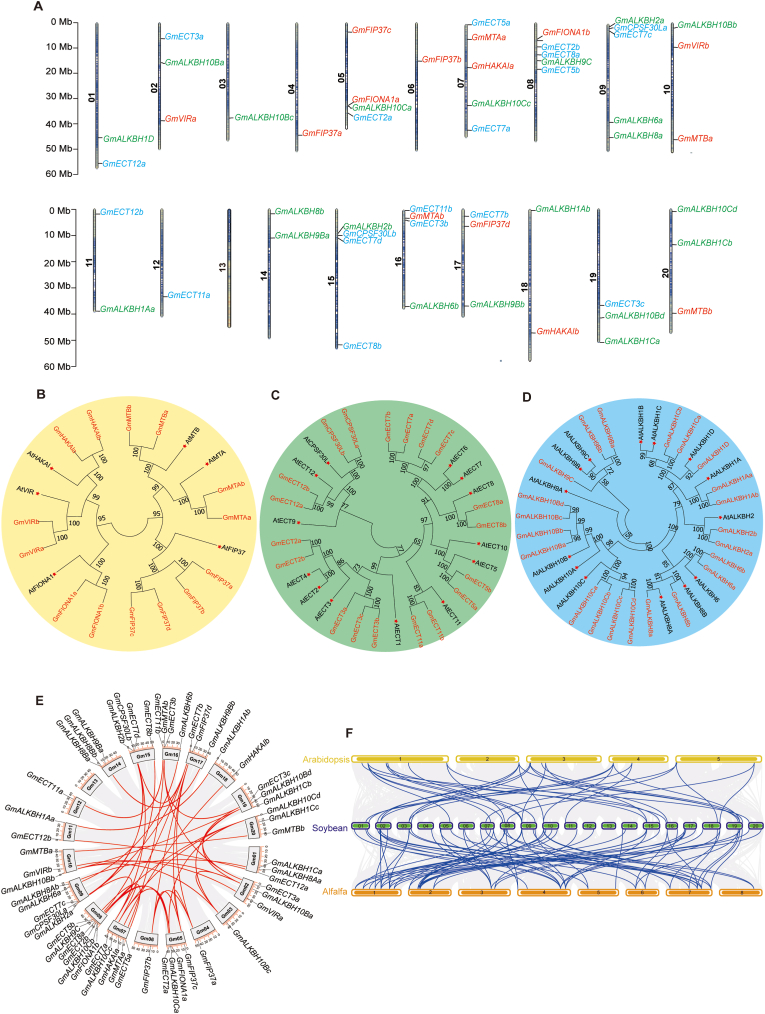
Genome-wide identification and characterization of m^6^A-associated genes in soybean. **A** Map showing the genomic localization of the 55 m^6^A regulatory genes identified in this study across 19 of the 20 chromosomes of the soybean genome. **B–D** Phylogenetic analysis of m^6^A-regulatory proteins based on 55 soybean proteins and 33 *Arabidopsis* proteins: writer components (**B**), reader components (**C**), and eraser components (**D**). **E** Intraspecific synteny analysis revealing collinearity patterns among m^6^A-related gene pairs, highlighting duplication events within the soybean genome. **F** Collinearity analysis of m^6^A-related genes among the *Arabidopsis*, soybean, and alfalfa genomes.

As an ancient tetraploid legume, soybean possesses numerous duplicated genes in its genome. In this study, we identified 55 putative m^6^A-regulatory genes through integrated BLAST and HMMER searches, using functionally characterized *Arabidopsis* homologs as queries. These genes belong to three functional categories: 14 writers, 22 erasers, and 19 readers. They are unevenly distributed across 19 of the 20 chromosomes of the soybean genome, the most genes (six) being located on chromosome 8, while chromosome 13 lacks any m^6^A-related gene (Fig. 1A). Physicochemical analysis of their encoded proteins revealed substantial variation among them (Table S1): GmALKBH2b is the shortest (125 amino acids [aa]), while VIRILIZER a (GmVIRa) is the longest (2,230 aa). Most of these proteins (72.7%) are acidic, with isoelectric points ranging from 5.08 (GmFIP37d) to 9.86 (GmALKBH2b). Over 75% (42/55) are predicted to be unstable based on an analysis on the ExPASy website, and all may be hydrophilic, as suggested by their negative grand average of hydropathy (GRAVY) values. Notably, GmFIONA1s may localize to chloroplasts, suggesting potential organellar functions.

To elucidate the evolutionary history of m^6^A regulatory genes in soybean, we performed phylogenetic reconstruction and comparative synteny analysis using MEGA 11. The phylogenetic trees incorporated m^6^A-regulatory proteins from soybean and *Arabidopsis*, revealing distinct evolutionary patterns among writers, erasers, and readers (Fig. 1B–D). Soybean exhibited substantial family expansion across m^6^A-related proteins. Soybean has four FIP37 homologs, while other writers are typically represented by two copies (Fig. 1B). Erasers showed distinct duplication patterns: GmALKBH10B and GmALKBH10C had four copies each, while GmALKBH1D, GmALKBH8A, and GmALKBH8B are represented by single copies (Fig. 1D). The GmECT2–4 subclade showed differential expansion, with two ECT2 and three ECT3 homologs. Notably, *ECT4* has no direct homologous genes in soybean, and this phenomenon may be associated with the functional redundancy of ECT2 and ECT3 (Fig. 1C)

To elucidate the evolutionary dynamics of m^6^A-related genes, we performed intra- and interspecies synteny analysis involving soybean, *Arabidopsis*, and alfalfa (*Medicago sativa*). We detected over 40 gene pairs present within collinear segments of the soybean genome, indicative of historical duplication events (Fig. 1E). Within the 55 genes analyzed, we identified 27 syntenic gene pairs (Table S2). With the exception of *GmALKBH1Ca* and *GmALKBH1Cb*, all duplicates originated from whole-genome or segmental duplications, underscoring the dominant role of large-scale duplication events in the expansion of the m^6^A gene family. To explore the selection pressure of the soybean m^6^A-related genes, we calculated the Ka and Ks substitution rates of all gene pairs, as well as their Ka/Ks values (Table S2). Ka/Ks analysis revealed purifying selection across all m^6^A-related gene pairs (Ka/Ks < 1), reflecting strong evolutionary constraints. Based on a synonymous substitution rate of 6.2 × 10^−9^ per site per year [29], we estimated the duplication events to have occurred between 3.40 million years ago (Mya) and 35.72 Mya, with most events occurring within the last 10 million years. There was a significant collinear correlation between the m^6^A-related genes in *Arabidopsis* and soybean, exhibiting a one-to-many relationship, which further indicates that the soybean m^6^A-related genes have undergone genome duplication events (Fig. 1F). The collinearity between m^6^A-related genes in alfalfa and soybean, both leguminous plants, was stronger than that between *Arabidopsis* and soybean, suggesting that the alfalfa genome also experienced duplication events.

### 2.2 Structural and regulatory features of m^6^A-associated genes

Structural analysis of the genes encoding the components of soybean m^6^A readers, writers, and erasers revealed that homologous gene pairs generally exhibit similar structural patterns (Fig. S1A). Most genes contain introns, although *GmVIRb* was distinct from its homolog *GmVIRa* due to its two large introns (>5 kb) resulting in a total genomic locus exceeding 25 kb. Motif analysis using MEME suite identified 10 conserved motifs across m^6^A proteins (Fig. S1B, Table S3). The writer components showed limited motif conservation, with only FIP37s and HAKAIs displaying consistent patterns. Conversely, for the eraser components, motif 5 was conserved across all members except GmALKBH2s, suggesting its evolutionary significance. Within the reader components, GmECTs consistently contained motifs 1, 2, and 4, which may represent key functional elements.

To identify potential upstream *cis*-regulatory elements, we analyzed the sequence of 2500-bp promoter fragments upstream of the start codon of each soybean m^6^A-related gene using the PlantCARE database. We identified over 10,000 putative *cis*-acting elements, averaging more than 190 elements per promoter. After filtering and classification (Table S4), we visualized selected elements along the promoters (Fig. S1C). The promoter regions contained binding elements for multiple regulatory pathways, including auxin-responsive elements, salicylic acid–responsive elements, and MYB-binding sites (MBS) (Fig. S1C and Table S4). Notably, we noticed the presence of numerous light-responsive elements (G-Box, Sp1, I-box, MRE), potentially linking m^6^A modification of RNA with photoperiod regulation and biological clock functions as reported in previous studies [30].

### 2.3 Analysis of tissue-specific expression and rhythmic expression in soybean under long-day conditions

**Fig. 2:**
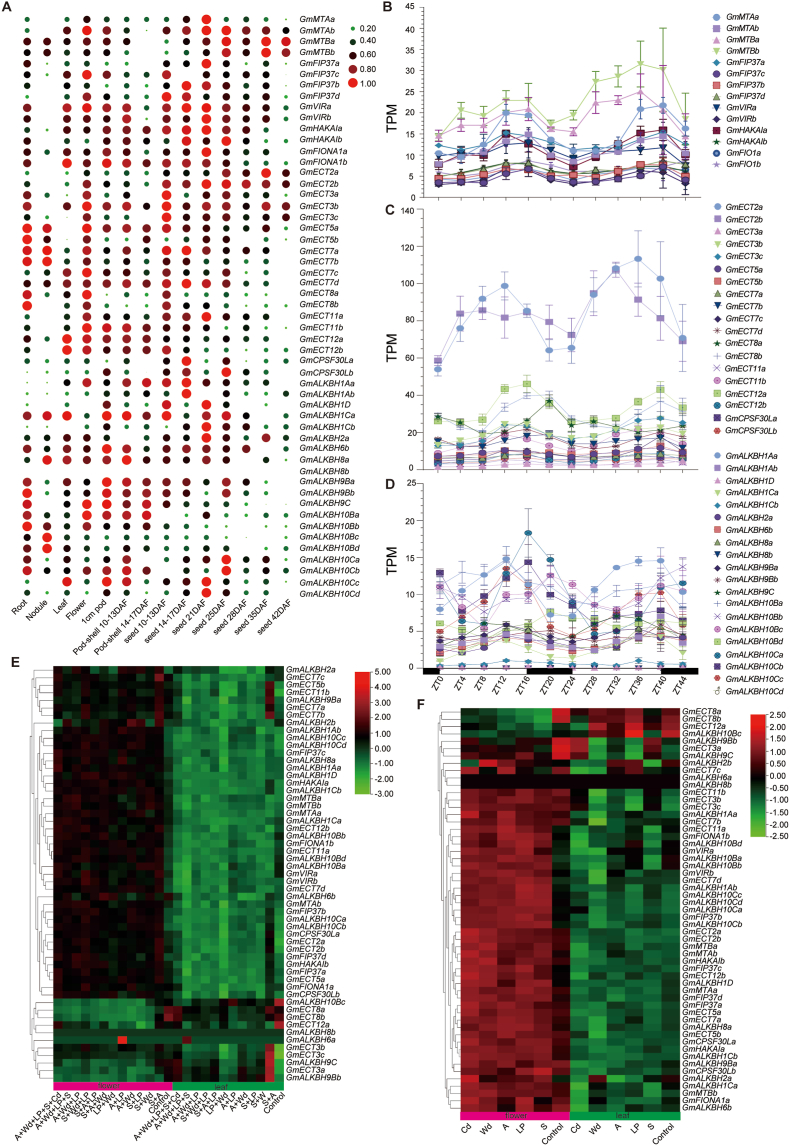
Tissue-specific and stress-responsive expression profiles of m^6^A regulatory genes in soybean. **A** Tissue-specific expression patterns across different soybean tissues. Circle diameter is proportional to expression intensity. **B–D** Temporal expression dynamics under long-day conditions over a 48-h time course for writer component genes (**B**), reader component genes (**C**), and eraser component genes (**D**). The white bars indicate subjective day, and the black bars subjective night. **E** Heatmap representation of transcript levels for m^6^A-related genes in flowers and leaves under combined stress treatments. Experimental conditions: CK (control), salt (S; 15 mM NaCl), low P (LP; 10% of normal phosphate levels), acidity (A; pH 4.0), water deficit (Wd; 30% of available transpiration water), and Cd (300 μM CdCl_2_). **F** Heatmap representation of transcript levels for m^6^A-related genes in flowers and leaves of plants subjected to individual stress conditions. All expression values are represented as Transcripts Per Million (TPM) and the data were log_2_-normalized during plotting to compare the expression trends of genes. DAF, days after flowering.

Expression analysis revealed the ubiquitous expression of m^6^A-related genes across tissues, with only *GmALKBH8b* showing no detectable expression (Fig. 2A), supporting a role for m^6^A modification throughout plant development. In nodules, writer genes were expressed at low levels (except *GmMTB*s) while they were highly expressed in roots. Previous studies documented the expression of writer genes during root development in *Arabidopsis* [31], suggesting that *GmMTB*s may influence both root development and nodule formation. Floral tissues displayed elevated expression levels of m^6^A-regulatory genes, highlighting m^6^A modification as a potential regulatory mechanism in flower development.

The plant circadian clock senses temporal and light cues. Given that *Arabidopsis* m^6^A regulatory genes display rhythmic expression, and considering the high photoperiod sensitivity of soybean plants, we investigated whether soybean m^6^A-regulatory genes respond to photoperiodic regulation. Analysis of publicly available transcriptome data revealed distinctive expression patterns for these genes (Fig. 2B–D). All genes exhibited bimodal expression with two peaks over the 48-h time course, suggesting diurnal expression; within homologous gene sets, one gene was typically expressed at higher level than the other gene. This pattern suggests both functional redundancy and potential compensatory effects among soybean m^6^A-related genes, similar to mechanisms observed for *GmFT2a* and *GmFT5a* for flowering time in soybean [32]. Further research is warranted to determine whether suppression of the primary gene in m^6^A-regulatory gene pairs triggers compensatory expression in the homolog.

Notably, the expression levels of the reader genes *GmECT2a* and *GmECT2b* were significantly higher than those of other Reader members (Fig. 2C), indicating that *GmECT2*s may play an important role in soybean. As homologs of *Arabidopsis* ECT2, a key player in m^6^A methylation recognition [7], this elevated expression of *GmECT2*s likely reflects their central function in detecting m^6^A modifications within the species.

### 2.4 Transcriptomic analysis of m^6^A genes in response to multifactorial stress

Global climate change increases the frequency of combined abiotic stresses, such as drought stress, salinity stress, heat, and nutrient deficiency, all severely limiting crop productivity. To investigate the response of m^6^A-related genes under such conditions, we analyzed their expression levels in soybean leaves and flowers under individual and combined stresses including salinity, low phosphorus, acidity, drought, and cadmium exposure. Consistent with their tissue-specific expression patterns, most m^6^A-related genes showed lower expression in leaves than in flowers (Fig. 2A, F). Under stress, their expression was largely downregulated in leaves and upregulated in floral tissues (Fig. 2A, F).

Multifactorial stress combinations (MFSCs), where plants face multiple simultaneous or sequential stressors, significantly threaten crop productivity [33]. Under MFSCs, m^6^A gene expression patterns mirrored those of the single stress responses: predominantly downregulation in leaves and upregulation in flowers (Fig. 2E and F). Notably, the expression of *GmALKBH9Bb* and *GmALKBH9C*, the soybean homologs of *AtALKBH9B* and *AtALKBH9C*, respectively, was induced in leaves under salinity stress and combined salinity–acid stress. These demethylases, known to mediate responses to ABA and heat stress [3,4], may contribute to soybean salinity tolerance and acclimation to MFSCs. Similarly, *SbALKBH9B* expression is induced under salinity and drought stress in sorghum [34], further suggesting a conserved role for these demethylases in stress response. The involvement of m^6^A-related genes in flowering regulation across species under abiotic stress supports a function in reproductive development in stress-challenged soybeans.

### 2.5 Direct interaction among m^6^A writer complex members in soybean

**Fig. 3:**
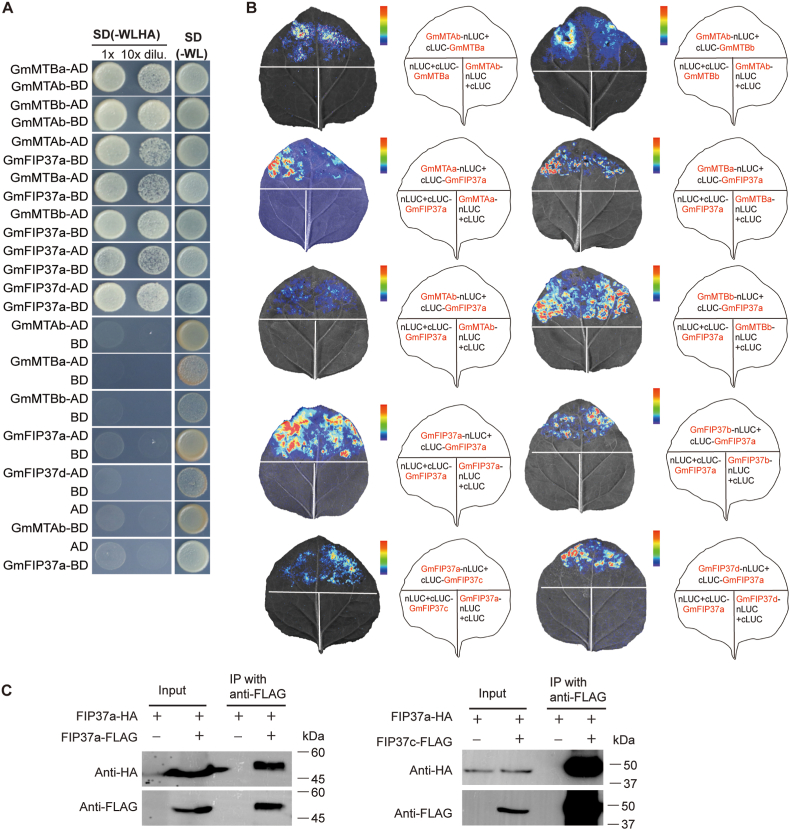
Molecular characterization of direct interactions within components of the soybean m^6^A writer complex. **A** Yeast two-hybrid analysis testing the protein–protein interactions among members of the m^6^A writer complex in soybean. Full-length coding sequences were cloned into vectors containing either the GAL4 activation domain (AD) or DNA-binding domain (BD). Yeast transformants were cultured on selective synthetic defined (SD) medium lacking tryptophan, leucine, histidine, and adenine (SD/−Trp/−Leu/−His/−Ade) to assess protein–protein interactions. Non-selective SD medium lacking tryptophan and leucine (SD/−Trp/−Leu) served as viability controls. **B** Protein–protein interaction assays among soybean m^6^A writer complex components via split-luciferase complementation assays in *Nicotiana benthamiana* leaves. Full-length GmMTAa/b, GmMTBa/b, and GmFIP37a/b/c/d were fused to either the N-terminal half (nLUC) or C-terminal half (cLUC) of firefly luciferase (LUC). Negative controls included co-infiltration of each gene*-nLUC* construct with the empty *nLUC* vector, and each gen*e-cLUC* construct with the empty *cLUC* vector. **C** Co-immunoprecipitation (Co-IP) assays confirming the specific *in vivo* interaction of FIP37a-HA with FIP37a-FLAG and of FIP37a-HA with FIP37c-FLAG. Co-IPs were conducted using total protein extracted from the leaves of 4-week-old *N. benthamiana* plants infiltrated with the corresponding constructs, followed by immunoblotting.

The m^6^A modification is a dynamic and reversible post-transcriptional modification of mRNA, primarily catalyzed by a multi-subunit writer complex. In Arabidopsis, this complex consists of MTA, MTB, and FIP37, and interacts with auxiliary proteins including VIR, HAKAI, HAKAI-INTERACTING ZINC FINGER PROTEIN 1 (HIZ1), and HIZ2 to form a functional regulatory unit [31]. Due to its tetraploid nature, soybean possesses multiple copies of genes homologous to these writers, suggesting a more complex interaction network. In this study, we systematically tested the interaction of proteins within the core GmMTA–GmMTB–GmFIP37 module using yeast two-hybrid and luciferase complementation imaging assays. We observed an interaction between GmMTBa and GmMTBb and the other components GmMTAb and GmFIP37a (Fig. 3A and B). Furthermore, co-immunoprecipitation (Co-IP) experiments confirmed that FIP37a forms both homodimers and heterodimers with its homologs, implying a mechanism underlying functional enhancement via oligomerization (Fig. 3C). Transcriptome profiling revealed that *GmFIP37a* is the most highly expressed *FIP37* homolog, supporting a role for the encoded protein in the assembly and stability of the m^6^A writer complex in soybean (Fig. 2B).

### 2.6 Histone modifications of m^6^A-related genes in soybeans

**Fig. 4:**
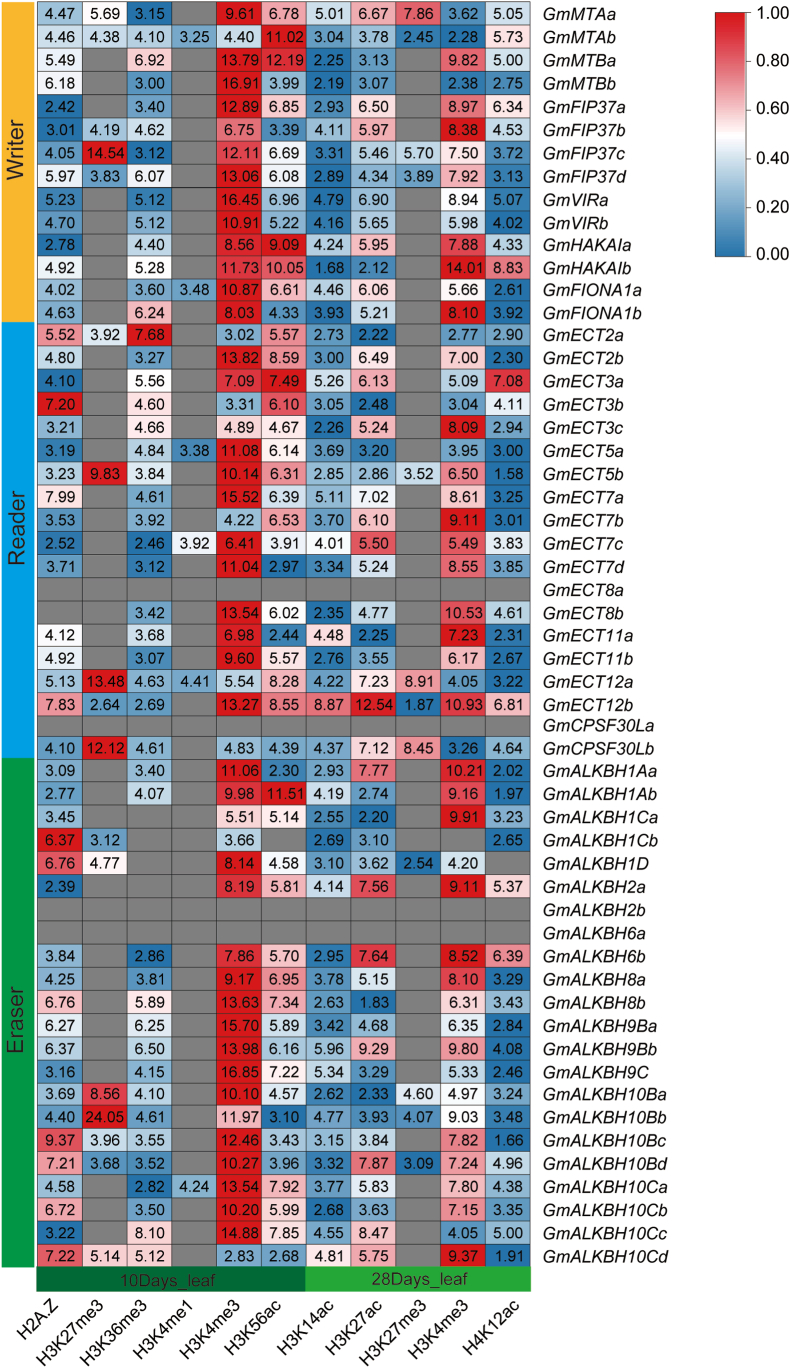
Histone modification patterns associated with m^6^A regulatory genes in soybean. The heatmap represents the fold enrichment values derived from ChIP-seq analysis. Color intensity represents modification levels: red regions indicate high fold enrichment, blue regions denote low fold enrichment; gray regions signify areas where no reliable data were obtained during ChIP-seq experiments. Numerical values within cells correspond to the calculated fold enrichment for each respective position.

Histone modifications represent a major epigenetic mechanism that regulates gene expression. To investigate their role in the transcriptional control of m^6^A-regulatory genes, we performed chromatin immunoprecipitation followed by deep sequencing (ChIP-seq) using the leaves of 10-day-old and 28-day-old soybean plants to produce genome-wide histone modification profiles (Fig. 4, S2, and Table S5). We determined that most m^6^A-related genes are associated with activating histone methylations such as H3K4me3 or H3K36me3, suggesting that histone methylation contributes to enhancing the expression levels of these genes. By contrast, we detected the repressive mark H3K27me3 only on a subset of genes. Notably, all *GmMTA* members, encoding the core components of the methyltransferase complex, were marked with H3K27me3, potentially inhibiting their expression, which might result in lower methylation levels of their target transcripts. Furthermore, the *GmFIP37c* locus harbored H3K27me3, as did its homolog *GmFIP37d* in the leaves of 28-day-old plants (Fig. 4, S2). This finding suggests that the expression of these genes may be inhibited during development. Besides histone methylation, we also measured the levels of acetylation marks (H3K56ac, H3K14ac, H3K27ac, H4K12ac) and H2A.Z modifications, which can either activate or suppress gene expression [35]. These findings collectively support a multi-layered epigenetic regulatory network modulating the expression of m^6^A-related genes in soybean.

### 2.7 Natural variation and selection of GmMTBs during domestication

**Fig. 5:**
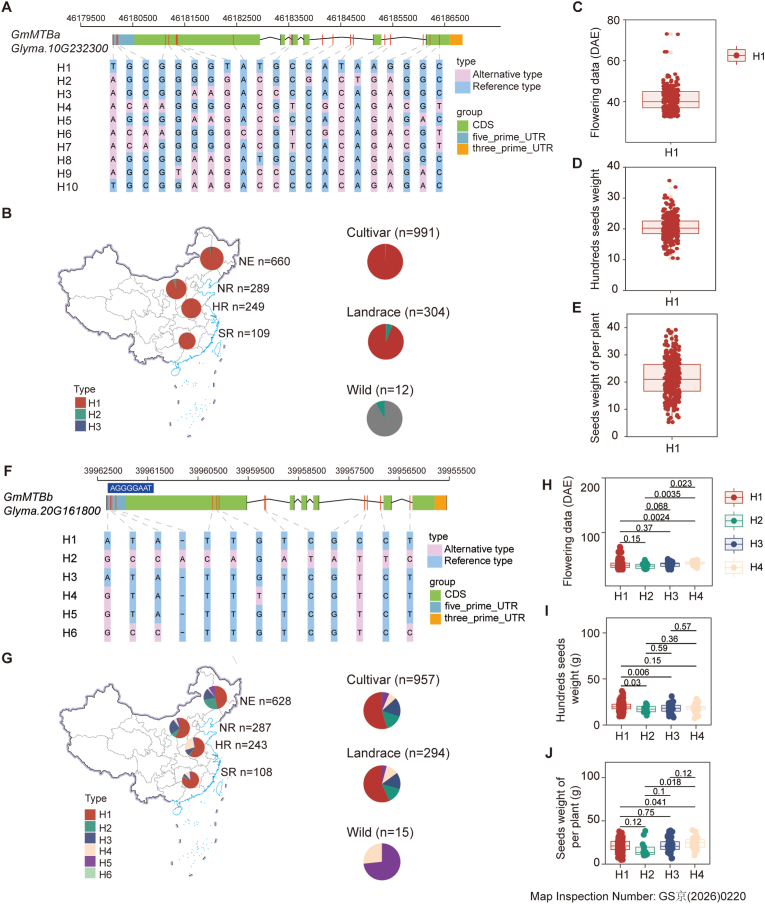
Haplotype analysis and agronomic trait associations of *GmMTB* genes in soybean. **A** Single nucleotide polymorphisms (SNPs) identified within the different haplotypes of *GmMTBa* across 1,495 soybean accessions. **B** Geographical distribution of *GmMTBa* haplotypes (H1–H10) across different regions of China, with pie charts representing relative frequencies. NE, Northeast region; NR, Northern region; HR, Huang-Huai-Hai region; SR, Southern region. **C-E** Key agronomic traits for the major *GmMTBa* H1 haplotype: days to flowering (**C)**, hundred-seed weight (**D)**, and grain yield per plant (E**)**. **F** SNPs identified within the different haplotypes of *GmMTBb*. **G** Geographical distribution of the *GmMTBb* haplotypes (H1–H6) across different regions of China, with pie charts representing relative frequencies. **H-J** Analysis of key agronomic traits among the four major *GmMTBb* haplotypes (H1–H4): days to flowering (**H)**, hundred-seed weight **(I)**, and grain yield per plant (**J)**. Box plot annotation: The box represents the interquartile range (IQR), spanning the 25th (Q1) to 75th (Q3) percentiles. The horizontal line inside the box indicates the median. Whiskers extend to the maximum and minimum values within 1.5-fold IQR, and scattered dots denote outliers.

To explore the potential role of m^6^A-related genes in soybean evolution, we performed haplotype analysis of the writer *GmMTB* genes across 1,495 accessions (Fig. 5, Table S6). Our analysis identified ten haplotypes for *GmMTBa* and six for *GmMTBb* (Fig. 5A, F).

Among the 10 haplotypes of *GmMTBa*, the haplotype Hap1 is present in over 95% of the cultivars, whereas the frequency of Hap2 progressively declined during domestication (Fig. 5B). This pattern suggests that Hap2 was selected for during soybean domestication. Notably, Hap1 is completely absent in wild soybean accessions, indicating that *GmMTBa* underwent strong selection pressure during breeding from wild to landraces. During early domestication, Hap1 was heavily favored by artificial selection, increasing in frequency from 0% in wild accessions to over 95% in cultivars, and has since maintained this high frequency in subsequent breeding programs (Fig. 5B). As a functionally significant gene, *GmMTBa* is evolutionarily conserved, with highly uniform sequences across germplasm resources. Consequently, *GmMTBa* is unlikely to further contribute to the phenotypic diversity of cultivated soybean populations (Fig. 5C–E).

For the *GmMTBa* homolog *GmMTBb*, represented by six haplotypes, H4 and H5 were consistently retained throughout breeding programs, from wild populations through landraces to cultivars, indicating positive selection throughout domestication and subsequent breeding. Cultivated soybean originated in the temperate Huang-Huai-Hai region in China, and its introduction to higher latitudes typically results in delayed flowering, often preventing full maturation within the growing season. We observed a possible signature of selection for the early-flowering haplotypes of *GmMTBb* (specifically *GmMTBb*^*H2/3*^) during adaptation to higher latitudes, allowing soybean to reach maturity before the arrival of winter (Fig. 5F, G and H). However, with the shortening of daylength, seed production diminishes significantly due to the shorter vegetative growth period and early flowering. The H4 haplotype *GmMTBb*^*H4*^ was present in wild accessions and may have been positively selected during domestication for adaptation to lower latitudes, resulting in a delayed flowering phenotype (Fig. 5H). This adaptation maintains optimal flowering periods in low-latitude regions, thereby ensuring stable seed production per plant (Fig. 5I and J). These findings demonstrate that m^6^A-related genes regulate the latitudinal adaptation of soybeans through modulation of flowering time, highlighting the intersection of epigenetic regulation and environmental adaptation during crop domestication.

### 2.8 GmMTBa-mediated m^6^A modification regulates salinity stress tolerance in soybean

**Fig. 6:**
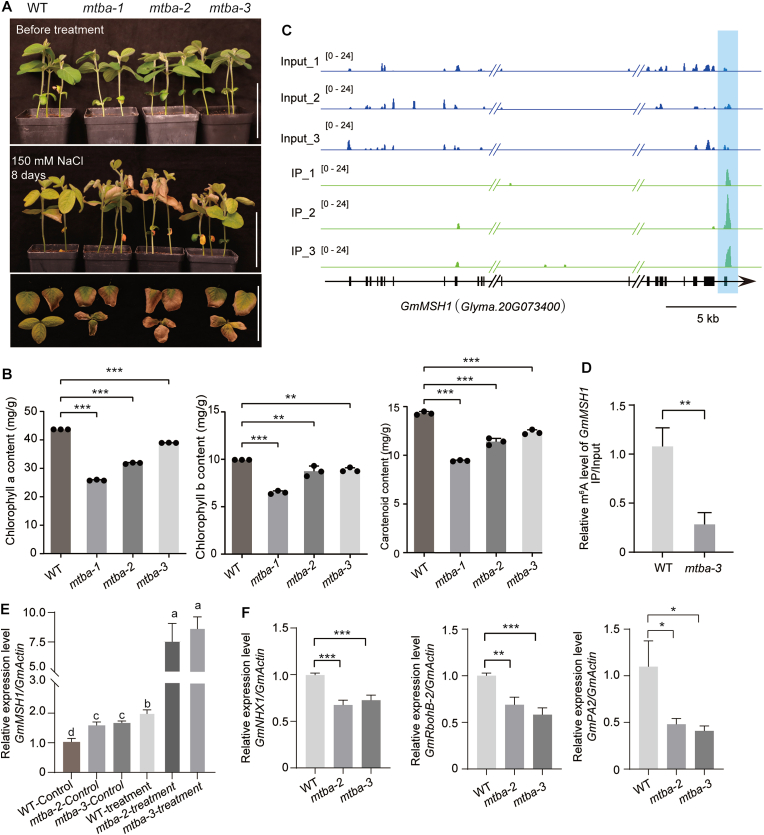
GmMTBa enhances salinity stress tolerance in soybean through m^6^A modification of transcripts from stress response genes. **A** Representative photographs of soybean plants after 8 days of exposure to 150 mM NaCl treatment. Scale bar, 10 cm. **B** Content of the photosynthetic pigments chlorophyll *a*, chlorophyll *b*, and carotenoids in four soybean lines following 8 days of salinity stress exposure. **C** Integrative Genomics Viewer visualization of RNA-seq reads mapping to the *GmMSH1* locus. Light blue box indicates the position of the m^6^A modification site in the *GmMSH1* mRNA. **D** m^6^A-IP-qPCR validation of m^6^A peaks in *GmMSH1* mRNA in wild-type (WT) and *Gmmtba* mutant plants. **E** Relative *GmMSH1* transcript levels in WT and *Gmmtba* mutant lines with or without salt treatment. Different lowercase letters indicate significant differences (*P* < 0.05) as determined by one-way analysis of variance (ANOVA) with Tukey's post hoc test. **F** Relative transcript levels of the salt stress–responsive genes *GmNHX1*, *GmRbohB-2*, and *GmPA2* in WT and *Gmmtba* mutant lines. In **(B–D,** and **F)**, values are means ± standard deviation (SD) from three biological replicates; statistical significance was determined using two-tailed Student's *t*-tests and is denoted as follows: ∗, *P* < 0.05; ∗∗, *P* < 0.01; ∗∗∗, *P* < 0.001.

Soil salinity represents a major environmental constraint on global crop productivity, affecting approximately 3% of global land area with increasing degradation of arable land due to salinization [36]. While the m^6^A modification has been implicated in plant responses to abiotic stress [25,37], its specific mechanisms in the tolerance to salinity stress in soybean remain incompletely understood. To investigate this question, we used gene editing to generate knockout mutants of *GmMTBa* and subjected the mutants to salinity stress. Upon irrigation with 150 mM NaCl, three independent *Gmmtba* mutant lines exhibited significantly more severe leaf senescence than wild-type plants, indicating that loss of GmMTBa function confers increased sensitivity to salinity stress in soybean (Fig. 6A). Quantification of photosynthetic pigment contents confirmed this phenotype, as mutant leaves contained substantially lower levels of chlorophylls and carotenoids under salinity stress (Fig. 6B). These findings complement previous reports demonstrating alkaline stress–induced expression of *GmMTBa* and enhanced alkaline stress tolerance following its transient overexpression [25], collectively establishing GmMTBa as a positive regulator of salinity stress responses in soybean.

To elucidate the molecular mechanism underlying GmMTBa-mediated salinity-stress tolerance, we took a closer look at our previously published m^6^A-seq data [24] to identify potential target transcripts of m^6^A modification. This search identified *GmMSH1* as a candidate m^6^A-modified target transcript (Fig. 6C). The *Arabidopsis* homolog of this gene functions as a key negative regulator of stress responses and plays a crucial role in stress signal transduction [26]. The regulation of *GmMSH1* by GmMTB has not been previously reported. m^6^A-IP-qPCR analysis confirmed the presence of the m^6^A modification on *GmMSH1* transcripts, with significantly lower methylation levels in *Gmmtba* mutants (Fig. 6D), indicating that the m^6^A modification of *GmMSH1* mRNA is dependent on GmMTBa*.* Expression analysis revealed significant upregulation of *GmMSH1* transcript levels under salinity stress (Fig. 6E). Elevated expression of the negative regulator of the stress signaling gene *GmMSH1* likely suppresses stress signal transduction, contributing to the salinity-stress sensitivity phenotype. Transcriptional profiling of the *Gmmtba* mutant lines and wild-type plants revealed significant downregulation of several established stress-responsive genes in the mutants. These differentially expressed genes included *Na*^*+*^*/H*^*+*^ *Antiporter 1* (*GmNHX1*), *Respiratory burst oxidase homolog B-2* (*GmRbohB-2*, associated with reactive oxygen species [ROS] generation) and *Peroxidase 2* (*GmPA2*). Previous studies have demonstrated that these genes positively regulate plant salinity-stress tolerance [36,38,39]. The substantial downregulation of these positive regulators of salinity-stress tolerance in the *Gmmtba* mutant lines confirms that loss of GmMTBa function disrupts normal regulation of stress response pathways (Fig. 6F).

**Fig. 7:**
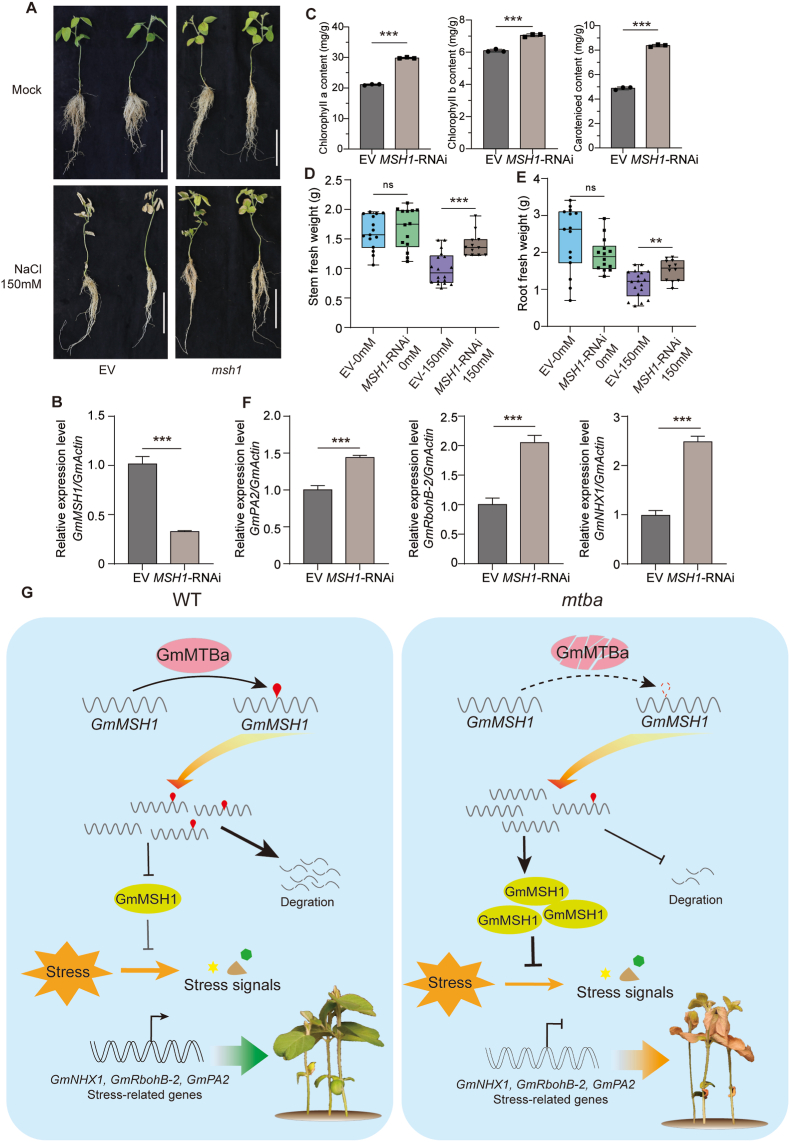
Proposed molecular mechanism underlying GmMTBa-mediated regulation of salinity stress responses in soybean. **A** Representative photographs of soybean plants exposed to 150 mM NaCl treatment or mock-treated with water only (Mock). EV, plants with hairy roots transformed with the empty vector; *MSH1*-RNAi, plants with hairy roots transformed with the RNA interference construct *MSH1*-RNAi. Scale bars, 10 cm. **B** Relative *GmMSH1* transcript levels in EV and *MSH1*-RNAi plants. **C** Contents of the photosynthetic pigments chlorophyll *a*, chlorophyll *b*, and carotenoids in the leaves of EV and *MSH1*-RNAi plants following 8 days of salinity stress. **D, E** Stem (**D**) and root (**E**) fresh weight of EV and *MSH1*-RNAi plants under different treatments. *n* ≥ 10. The box represents the interquartile range (IQR), the middle horizontal line indicates the median, and the whiskers extend to the maximum and minimum values. **F** Relative transcript levels of salinity stress–responsive genes in EV and *MSH1*-RNAi plants treated with 150 mM NaCl: *GmNHX1*, *GmRbohB-2*, and *GmPA2*. In (**B–F**), values are means ± SD. from three biological replicates. Statistical significance was determined using two-tailed Student's *t*-tests: ∗, *P* < 0.05; ∗∗, *P* < 0.01; ∗∗∗, *P* < 0.001. **G** Proposed molecular mechanism underlying GmMTBa-mediated regulation of salinity stress responses in soybean. In wild-type plants, GmMTBa facilitates m^6^A methylation of *GmMSH1* transcripts, promoting their degradation and enabling effective stress signal transduction, which ultimately confers tolerance to salinity stress. Conversely, in *Gmmtba* mutant plants, diminished m^6^A methylation of *GmMSH1* mRNA results in transcript stabilization and accumulation, leading to attenuated stress signaling. This dysregulation causes aberrant expression of downstream stress-responsive genes, manifesting phenotypically as higher sensitivity to salinity stress.

To further elucidate the biological function of GmMSH1, we generated *GmMSH1*-knockdown hairy-root plants (designated *MSH1*-RNAi) via RNA interference (RNAi). RT-qPCR confirmed a significant drop in *GmMSH1* transcript levels in these plants compared to the empty vector (EV) control (Figs. 7A, B, S3). When subjected to 150 mM NaCl treatment, the *MSH1*-RNAi plants were more tolerant to salinity stress, a phenotype opposite that of the *Gmmtba* mutants (Fig. 7A). The *MSH1*-RNAi plants retained significantly higher chlorophyll contents and had higher shoot and root fresh weights than the EV controls under salinity stress (Fig. 7C–E). These results indicate that GmMSH1 acts as a negative regulator of the soybean salinity stress response. Consistent with this proposed role, expression levels of the salinity tolerance–associated genes *GmPA2*, *GmRbohB-2*, and *GmNHX1* were significantly higher in the *MSH1*-RNAi plants (Fig. 7F), supporting the observed enhancement of salinity-stress tolerance.

Based on these findings, we propose a model for GmMTBa-mediated salinity-stress tolerance in soybean (Fig. 7G). In wild-type plants, GmMTBa directs the m^6^A modification of *GmMSH1* mRNA, promoting its degradation and thus maintaining low *GmMSH1* transcript levels. This post-transcriptional regulation facilitates proper stress signal transduction and enables appropriate expression of downstream stress-responsive genes, collectively contributing to salinity-stress tolerance. By contrast, loss of GmMTBa function results in lower m^6^A modification of *GmMSH1* mRNA, leading to transcript stabilization and accumulation. Elevated levels of translated GmMSH1 subsequently suppress stress signal transduction and inhibit the activation of downstream stress-responsive genes, ultimately resulting in heightened sensitivity to salinity stress. This model establishes a direct mechanistic link between epitranscriptomic regulation and abiotic stress acclimation in soybean, highlighting the role of the m^6^A modification in conferring agricultural stress resilience.

## 3 Discussion

RNA methylation, particularly the N^6^-methyladenosine (m^6^A) modification, represents a critical class of epitranscriptomic modifications regulating RNA translation, splicing, stability, and transport [40]. This dynamic and reversible modification has been extensively characterized in model organisms, but its role in crop species has been less explored. Leveraging advances in high-throughput sequencing technologies and comparative genomics, we conducted a comprehensive analysis of the m^6^A regulatory machinery in soybean to elucidate its biological significance and functional implications.

Our genome-wide analysis identified 55 m^6^A-regulatory genes scattered across the soybean genome, encompassing genes encoding m^6^A writers (methyltransferases), readers (binding proteins), and erasers (demethylases) (Fig. 1B–D). Within the writer complex, we identified four FIP37 homologs in soybean, with subsequent interaction and expression analyses suggesting *GmFIP37a* as the primary functional homolog (Fig. 1, Fig. 2A, B). This observation aligns with previously documented genetic compensation mechanisms in soybean, such as those observed for *GmFT2a* and *GmFT5a* [32], suggesting that secondary homologs may upregulate their expression to maintain essential functions when the primary homolog is compromised. Furthermore, our protein–protein interaction studies demonstrated that GmFIP37a can form homodimers, potentially enhancing its catalytic activity, while heterodimer formation among GmFIP37 paralogs may facilitate recognition of diverse target sites. Furthermore, several non-canonical m^6^A-binding proteins, such as RNA-binding protein 33 (RBM33) and members of the Insulin like growth factor 2 mRNA binding protein (IGF2BP) family, have been characterized in animals. To date, however, definitive homologs for these proteins, as well as of the demethylase Fat mass and obesity-associated (FTO), have not been identified in *Arabidopsis*. It is possible that plant genomes may encode functionally analogous proteins with sequence similarity to these animal factors. Notably, the soybean genome comprises approximately 54,000 genes, substantially more than the roughly 25,000 genes present in the Arabidopsis or human genomes. This expanded gene repertoire raises the possibility that candidate orthologs or functional analogs to these non-canonical m^6^A regulators might exist in soybean. Any such candidates would require functional validation to determine whether they perform m^6^A-related roles comparable to those of their animal counterparts.

The evolutionary history of legumes, comprising two subgenera that diverged approximately 10 Mya [41], provides important insights for understanding the genomic architecture of m^6^A writers, readers, and erasers. Recent advances in legume genomics, including the construction of a pan-genome for the subgenus *Glycine* [41,42], have significantly enhanced our understanding of soybean genome evolution. Historical evidence indicates that leguminous plants experienced an ancient polyploidization event approximately 65 Mya, followed by a more recent whole-genome duplication specifically within the *Glycine* lineage within the past 10 million years [42]. Our molecular dating analysis revealed that the divergence of m^6^A-related gene pairs in soybean predominantly occurred 3–10 Mya, consistent with the timeframe of the recent whole-genome duplication event, suggesting that the current complement of m^6^A regulatory genes largely arose from this duplication. Comparative genomic analysis between annual and perennial soybean species uncovered a loss of approximately 70% of all m^6^A-related loci in annual varieties [42], raising intriguing questions regarding the potential role of the m^6^A modification machinery in regulating meristematic activity and growth habit. Future investigations should examine whether specific m^6^A-related genes have been differentially retained or lost between annual and perennial soybeans, and whether such differences contributed to the evolutionary transition from the perennial to the annual growth strategy.

Domestication imposes strong directional selection on crop species, culminating in lower genetic diversity and declines in population polymorphisms toward homogenization. In this study, we discovered that both *GmMTB*s have undergone selection during soybean domestication (Fig. 5). A single haplotype of *GmMTBa* is nearly fully fixed in cultivars (Fig. 5B), indicative of intense selective pressure and suggesting that it likely will contribute little to future phenotypic differentiation in elite germplasm. By contrast, *GmMTBb* displayed clear haplotype divergence (Fig. 5F). To assess whether sequence variation in the 5′ untranslated region (5' UTR) among *GmMTBb* haplotypes contributes to their phenotypic differences, we performed dual-luciferase reporter assays (Fig. S4A). These assays showed that 5′ UTR variation does not significantly affect translational efficiency (Fig. S4B), suggesting that the phenotypic differences are more likely attributable to variation in the coding sequence or distal promoter regions of the *GmMTBb* haplotypes, possibilities that require experimental testing.

To explore the basis of the functional divergence between GmMTBa and GmMTBb, we analyzed their conserved domains and predicted 3D structures. Both proteins contain a conserved MT-A70 domain, confirming their membership to the methyltransferase family (Fig. S4C and D). However, substantial sequence variation in their N-terminal domains likely reflects their differential selection during domestication. Predictions of their 3D structures further revealed distinct spatial conformations between GmMTBa and GmMTBb (Fig. S4E–G), supporting the hypothesis that GmMTBb may perform unique biological functions. Analysis of sequence variation within the coding regions revealed the presence of multiple single nucleotide polymorphisms (SNPs). However, among haplotypes represented by at least three accessions, non-synonymous changes leading to altered amino acids were only observed between *GmMTBa*^*H2/H4*^ and *GmMTBa*^*H1*^ (note that *GmMTBa*^*H2*^ and *GmMTBa*^*H3*^ encode identical proteins), as well as between *GmMTBb*^*H2/H4*^ and *GmMTBb*^*H1*^ (Fig. S5A and E). Predictions of the 3D structures for the proteins encoded by these haplotypes indicated that all proteins retained a conserved MT-A70 domain with a remarkably conserved structure (Figs. S4E and S5). By contrast, amino-acid sequence variations were associated with conformational changes primarily in peripheral regions surrounding the core MT-A70 methyltransferase domain (Fig. S5). These structural differences likely contribute to functional divergence among proteins encoded by different haplotypes, potentially affecting protein–protein interactions or RNA-binding specificity toward target genes.

In *Arabidopsis*, the blue light–mediated interaction between FIP37 and cryptochrome 1 (CRY1) plays a critical role in photomorphogenesis [40]. Our investigations confirmed the conservation of core interactions within components of the writer complex in soybean, suggesting functional conservation across species. However, the polyploid nature of soybean has resulted in an expansion of homologous genes, raising the possibility of functional divergence or redundancy among GmFIP37s and their interacting partners, particularly with soybean CRY1 homologs. Soybean also has homologs of Arabidopsis FIONA1, which regulates chlorophyll homeostasis through blue light–dependent phase separation via interactions with CRY2 and SUPPRESSOR of PHYTOCHROME A (SPA1) [43]. The soybean FIONA1 homologs were predicted to localize to chloroplasts, suggesting that they may fulfill similar functions through interactions with CRY2 homologs. The reader proteins ECT2–4, which form trimers and recruit Protein in chloroplast ATPase biogenesis (PAB) proteins in response to ABA during seed development in Arabidopsis [5], have homologs in soybean, with the notable exception of ECT4, which may have been lost due to functional redundancy with ECT2 and/or ECT3 during soybean evolution [5,41].

Histone modifications serve as powerful epigenetic regulators of gene expression by modulating chromatin accessibility and transcriptional activity. Previous studies have established that H3K4me3, H3K36me3, and histone acetylation are activating marks that enhance transcription. Our genomic analysis revealed that the majority of soybean m^6^A-related loci (over 90%, 51/55) exhibit one or more of these activating modifications (Fig. 4, S2 and Table S5), indicating that transcriptional regulation of the m^6^A modification machinery involves complex epigenetic mechanisms. Furthermore, nearly all m^6^A-regulatory genes were marked by H2A.Z deposition, with a subset also displaying H3K27me3 modifications, both associated with transcriptional repression in Arabidopsis [35]. This observation warrants further investigation to determine whether a potential synergistic interaction between H2A.Z and H3K27me3 might mediate transcriptional repression of these genes at specific developmental stages or under different environmental conditions.

The m^6^A modification system plays essential roles in plant reproduction, as mutations in any component of the core MTA–MTB–FIP37 complex result in embryonic death in *Arabidopsis* [17]. FIP37 specifically regulates the determination of stem cell fate in *Arabidopsis* [44]. In rice, the FIP37 homolog OsFIP37 influences microspore formation through m^6^A modification of transcripts translated into threonine proteases and nucleoside-triphosphatases (NTPases) [44], while also promoting auxin biosynthesis in pollen mother cells, thereby regulating male meiosis and ultimately affecting grain yield [14]. However, the functional characterization of GmFIP37s, their potential functional redundancy, and their specific roles in the reproductive development of soybean remain to be elucidated. We are currently employing clustered regularly interspaced short palindromic repeats (CRISPR)/CRISPR-associated nuclease 9 (Cas9)-mediated gene editing to systematically investigate the regulatory mechanisms by which these proteins influence soybean yield and flowering time.

Our previous research demonstrated that GmMTBa, a core subunit of the soybean m^6^A methyltransferase complex, modulates global m^6^A modification levels and regulates plant height [24], similar to the functions of GmMTA family members [18]. Notably, while *GmMTA2* expression is also stress-inducible, the transient overexpression of this gene does not enhance soybean tolerance to abiotic stresses [25]. In the present study, we observed that *Gmmtba* mutant lines display pronounced sensitivity to salinity stress (Fig. 6A). This phenotypic evidence shows the role of GmMTBa in conferring abiotic stress tolerance in soybean. Notably, NaCl treatment significantly elevated *GmMSH1* transcript levels in *Gmmtba* mutants, suggesting that mutation of *GmMTBa* raises *MSH1* expression and consequently suppresses stress signal transduction. Furthermore, we identified *GmMSH1* transcripts as direct targets of GmMTBa-mediated m^6^A modification (Fig. 6C and D). Collectively, these findings establish that GmMTBa contributes to abiotic stress acclimation by negatively regulating the expression of the stress signaling suppressor gene *GmMSH1* through m^6^A modification (Fig. 7G). This discovery provides mechanistic insights into epitranscriptomic regulation of stress responses in soybean and establishes a framework for further investigation of m^6^A-mediated acclimation to environmental challenges in crop species.

In summary, we identified 55 m^6^A regulatory genes in soybean, phylogenetically classified as writers, readers, or erasers. Protein–protein interaction analysis confirmed the assembly of conserved core GmMTA-GmMTB-GmFIP37 writer complexes, with GmFIP37s exhibiting both homodimerization and heterodimerization capabilities. Notably, domestication-selected *GmMTBa*, encoding a core methyltransferase subunit, conferred salinity-stress tolerance by mediating m^6^A-dependent degradation of *GmMSH1* transcripts, a key negative regulator of stress signaling. These findings provide a foundation for understanding the regulation of the m^6^A modification in soybeans and position *GmMTBa* as a promising target for enhancing salinity-stress resilience in soybean.

## 4 Materials and methods

### 4.1 Plant materials and growth conditions

The soybean (*Glycine max* L. Merr.) cultivar ‘Williams 82’ served as the primary experimental material in this study. Seeds were sown in pots containing a nutritional soil mixture (LV12625, Pindstrup) of matrix and vermiculite (3:2, v/v). Plants were cultivated under controlled environmental conditions in a growth chamber maintained at 26 °C under a 16-h light/8-h dark photoperiod with 50% relative humidity. Three distinct CRISPR/Cas9-edited mutants, designated as *Gmmtba-1*, *Gmmtba-2*, and *Gmmtba-3* (denoted as *mtba-1/2/3* in the figures), were previously characterized and used in this study [24]. Seeds of Williams 82 and the *Gmmtba* mutants were germinated in vermiculite and subjected to 150 mM NaCl treatment at the V1 stage (first trifoliolate stage). After 5 days of treatment, leaf tissues were harvested, immediately flash-frozen in liquid nitrogen, and stored at −80 °C for subsequent RNA extraction. Phenotypic evaluations were conducted following 8 days of treatment.

To generate *GmMSH1* knockdown lines via RNA interference, a 250-bp specific fragment from the 5′ region of *GmMSH1* (+1 bp to +250 bp relative to the ATG start codon) was cloned into the pG2RNAi2 vector [45]. Hairy-root transformation was performed as follows: surface-sterilized soybean seeds were germinated on kraft paper; after germination, hypocotyls were excised to remove roots while retaining the shoots. *Agrobacterium rhizogenes* strain K599 carrying the RNAi construct was applied to the wound sites to induce hairy-root formation. Successful transformation was first confirmed by GFP fluorescence, followed by RT-qPCR to verify knockdown efficiency. Plants with positively transformed roots were transferred to vermiculite and grown for 3 days before being subjected to salinity stress via irrigation with 150 mM NaCl. On day 8 of stress treatment, leaves were collected for chlorophyll measurement. When wilting symptoms appeared, roots were harvested, flash-frozen in liquid nitrogen, and stored at −80 °C for subsequent RNA extraction. Whole-plant fresh weight was also measured at this stage.

### 4.2 Identification of putative m^6^A-regulatory genes in the soybean genome

The reference genome and annotation files for the soybean cultivar Williams 82 (Wm82.a4.v1) were retrieved from the Soybase database (https://soybase.org/). The sequences of *Arabidopsi*s m^6^A-related proteins were obtained from the TAIR database (https://www.arabidopsis.org/), along with the corresponding genome and annotation files. The alfalfa genome data and annotation files were acquired from the EnsemblGenomes database (https://ftp.ensemblgenomes.ebi.ac.uk/pub/plants/release-57). To comprehensively identify homologs of m^6^A-related genes in soybean, multiple approaches were employed. First, *Arabidopsis* protein sequences were used as queries against the soybean genome hosted at the Phytozome database (https://phytozome-next.jgi.doe.gov/). Concurrently, HMMER searches were performed using the 2OG-FeII_Oxy domain (cl21496) and YTH domain (PF04146) to identify additional homologous genes in soybean. The resulting sequences were compiled and filtered to eliminate redundant entries. Final validation of the putative m^6^A-related genes in soybean was conducted using the CD-Search program from the NCBI database for the presence of functional domains.

### 4.3 Structural characterization and evolutionary analysis of m^6^A-regulatory genes in soybean

Gene structural information and chromosomal locations for the m^6^A-regulatory genes identified in the soybean genome were extracted from the soybean genome annotation file and visualized with TBtools software [46]. The m^6^A methyltransferases (writers), demethylases (erasers), and m^6^A-binding proteins (readers) were separately subjected to phylogenetic analysis using MEGA-X, based on their relationships with their homologous *Arabidopsis* m^6^A-related genes. Phylogenetic trees were reconstructed using the neighbor-joining method with 1,000 bootstrap replicates, while other parameters were maintained at default settings.

Putative *cis*-acting regulatory elements were identified by analyzing 2500-bp promoter sequences upstream of the translation start codon of each soybean m^6^A-related gene using PlantCARE software [47]. Conserved protein motifs were characterized using the MEME suite (https://meme-suite.org/meme/) with the following parameters: any number of repetitions, maximum of 10 motifs, and optimum motif widths ranging from 6 to 100 amino acid residues. The spatial distribution of identified motifs within proteins was visualized using TBtools.

Duplication patterns and syntenic relationships among m^6^A-related genes in soybean, *Arabidopsis*, and alfalfa were analyzed using MCScanX and visualized with TBtools. The ratio of non-synonymous (Ka) to synonymous (Ks) nucleotide substitution rates was calculated to evaluate selection pressure acting on duplicated gene pairs. Divergence times between paralogous and orthologous gene pairs were estimated following the methodology described previously [29].

### 4.4 Transcriptome profiling and molecular characterization of m^6^A regulatory components

Tissue-specific expression profiles of m^6^A-regulatory genes were analyzed using RNA-seq data obtained from Soybase (https://legacy.soybase.org/soyseq/tables_lists/index.php?p&equals;ontology), encompassing diverse tissues including roots, nodules, leaves, flowers, pods, and seeds. Transcript abundance was quantified as Transcripts Per Kilobase of exon model per Million mapped reads (TPM) values and visualized via heatmap of TBtools. Diurnal expression patterns of m^6^A-related genes were examined using publicly available data from the SRA database (accession number PRJNA369113), with temporal expression profiles plotted using GraphPad Prism 9. To investigate transcriptome responses to abiotic stressors, public RNA-seq datasets from the NCBI database were analyzed (accession number GSE237798) [33]. The resulting stress-responsive expression patterns were visualized using TPM values and heatmaps generated with TBtools.

Total RNA was extracted from samples using an Eastep™ Super Total RNA Extraction Kit (Promega) according to the manufacturer's protocol. RNA quantity and quality were assessed using a NanoDrop spectrophotometer (Thermo Scientific, USA). First-strand cDNA synthesis was performed using a SPARKscript II RT Plus Kit with gDNA Eraser (SparkJade), starting with 800 ng of total RNA as template. The full-length coding sequences encoding components of the m^6^A writer complex were amplified from the resulting cDNA by PCR using PrimeSTAR® Max DNA Polymerase (2X, TaKaRa) with gene-specific primers as detailed in Table S7.

### 4.5 Protein–protein interaction analysis

The GAL4-based yeast two-hybrid system vectors pGADT7 and pGBKT7 were employed for yeast-based interaction assays. For luciferase complementation imaging (LCI) assays, the vectors pCAMBIA1300-35S-nLUC and pCAMBIA1300-35S-cLUC were used. All constructs were generated by inserting the full-length coding sequences of the respective genes into each vector backbone by enzyme-based cloning. The *nLUC/cLUC* fusion constructs were introduced into *Agrobacterium tumefaciens* strain GV3101 and positive colonies were co-infiltrated in the leaves of 4-week-old *Nicotiana benthamiana* plants. Additionally, the full-length sequences of *GmFIP37a* and *GmFIP37c* were cloned in-frame and upstream of the sequence encoding the HA or Flag tag and downstream of the CaMV 35S promoter; the resulting constructs were introduced into Agrobacterium strain GV3101 and positive colonies were co-infiltrated in the leaves of 4-week-old *N. benthamiana* plants. Proteins were extracted with extraction buffer (50 mM Tris-Hcl [pH 7.5], 150 mM NaCl, 1 mM EDTA, 0.1% SDS, 1 mM PMSF, and 1X Complete Protease Inhibitor Cocktail). Protein complexes were enriched using Flag magnetic beads (Thermo Scientific, A36798, 1:200 dilution), followed by immunoblotting with anti-Flag (Sigma, A8592, 1:2000 dilution) and anti-HA (Roche, 12013819001, 1:2000 dilution) antibodies.

### 4.6 Analysis of histone modifications

Chromatin immunoprecipitation followed by deep sequencing (ChIP-seq) data for leaf tissues collected from 10-day-old and 28-day-old Williams 82 plants were obtained from the Soybean Multi-omics Database (SoyMD, https://yanglab.hzau.edu.cn/SoyMD/#/) [48]. Analysis focused on the 3-kb genomic regions flanking m^6^A-related genes, with fold enrichment values used as indicators of histone modification status. Enrichment patterns were visualized as heatmaps using TBtools software. Chromosome-scale visualization of epigenetic features was performed using PyGenomeTracks v3.9 [49].

### 4.7 Haplotype analysis

Sequencing data for 1,495 soybean accessions and their corresponding phenotypic information were downloaded from the Soybean Multi-omics Database (SoyMD, https://yanglab.hzau.edu.cn/SoyMD/#/). SNP haplotype analysis and phenotypic correlations were conducted and visualized using the R package geneHapR [50].

### 4.8 Measurement of photosynthetic pigments

The contents of total chlorophyll and carotenoids were quantified using an acetone extraction–based protocol [51]. Briefly, fresh tissue was homogenized in 80% (v/v) aqueous acetone, subjected to a brief static extraction, and then adjusted to final volume with the same solution for spectrophotometric analysis. Absorbance values were measured at 663 nm, 646 nm, and 470 nm. Pigment concentrations were calculated as follows: chlorophyll *a* (C_*a*_, mg/L) = 12.21 × A663 − 2.81 × A646; chlorophyll *b* (C_*b*_, mg/L) = 20.13 × A646 − 5.03 × A663; and total carotenoids (C_x + c_, mg/L) = (1000 × A470 − 3.27 × C_*a*_ − 104 × C_*b*_)/229. The final pigment content (mg/g fresh weight [FW]) was calculated as (concentration × extraction volume × dilution factor)/sample weight. Measurements were based on three leaves per genotype with three biological replicates.

### 4.9 m^6^A immunoprecipitation and quantitative PCR (m^6^A-IP-qPCR) analysis

m^6^A-IP-qPCR was performed as previously described [52]. Briefly, 10 μg of total RNA was subjected to sonication for fragmentation, followed by pre-clearing with Protein A/G magnetic beads (Vazyme, PB101, 1:50 dilution). One-tenth of the sample was reserved as input control, while the remaining RNA was immunoprecipitated using m^6^A-specific antibodies (Abcam, ab151230, 1:200 dilution). The immunoprecipitated RNA and input samples were reverse-transcribed using HiScript III RT SuperMix for qPCR (Vazyme, R323-01) according to the manufacturer's instructions. Relative m^6^A methylation levels were quantified by quantitative PCR using SuperStar Blue Universal SYBR Master Mix (CWBIOCWBIO, CW3390H). All experiments were performed with three independent biological replicates and three technical replicates. Primer sequences used for qPCR analysis are provided in Table S7.

### 4.10 Dual-luciferase reporter assays

The sequence upstream of the *GmMTBb* start codon was cloned from the accession W82 harboring the Hap1; the corresponding Hap4 sequence was generated via site-directed mutagenesis of the Hap1 sequence. Homologous recombination was then performed using the BamHI and HindIII restriction sites to construct the recombinant plasmids pGreenII-0800-*GmMTBb*^*H1*^*:LUC* and pGreenII-0800-*GmMTBb*^*H4*^*:LUC*, which were introduced into *Agrobacterium* strain GV3101. Positive colonies were infiltrated into the leaves of 4-week-old *N. benthamiana* plants. A Dual Luciferase Reporter Gene Assay Kit (Beyotime, RG027) was used to detect firefly luciferase (LUC) and *Renilla* luciferase (REN) activities with a 96-well plate (Corning, 3917) in accordance with the manufacturer's instructions.

## CRediT authorship contribution statement

**Leili Wang:** Writing – original draft, Visualization, Validation, Data curation. **Chengyang Song:** Methodology, Data curation. **Zhu Yan:** Validation, Data curation. **Tianqi Wang:** Visualization, Validation, Methodology. **Yisheng Fang:** Methodology, Formal analysis. **Xiulin Liu:** Investigation, Formal analysis. **Junlong Bao:** Software. **Dan Zhu:** Writing – review & editing, Resources. **Xiao Luo:** Writing – review & editing, Resources, Project administration, Methodology, Funding acquisition, Conceptualization.

## Declaration of competing interest

The authors declare that they have no known competing financial interests or personal relationships that could have appeared to influence the work reported in this paper.

## Data Availability

All data generated or analyzed during this study are included in this published article and its supplementary information files.
