# Supplementary material for: Comprehensive analysis of m6A-regulatory genes in soybean uncovers GmMTBa as a critical determinant of salinity stress tolerance
Source: aBIOTECH. 2026 Mar 28;7(2):100046. doi: 10.1016/j.abiote.2026.100046 (PMC13096960; doi:10.1016/j.abiote.2026.100046)
Supplement: Multimedia component 1 [file mmc1.docx]

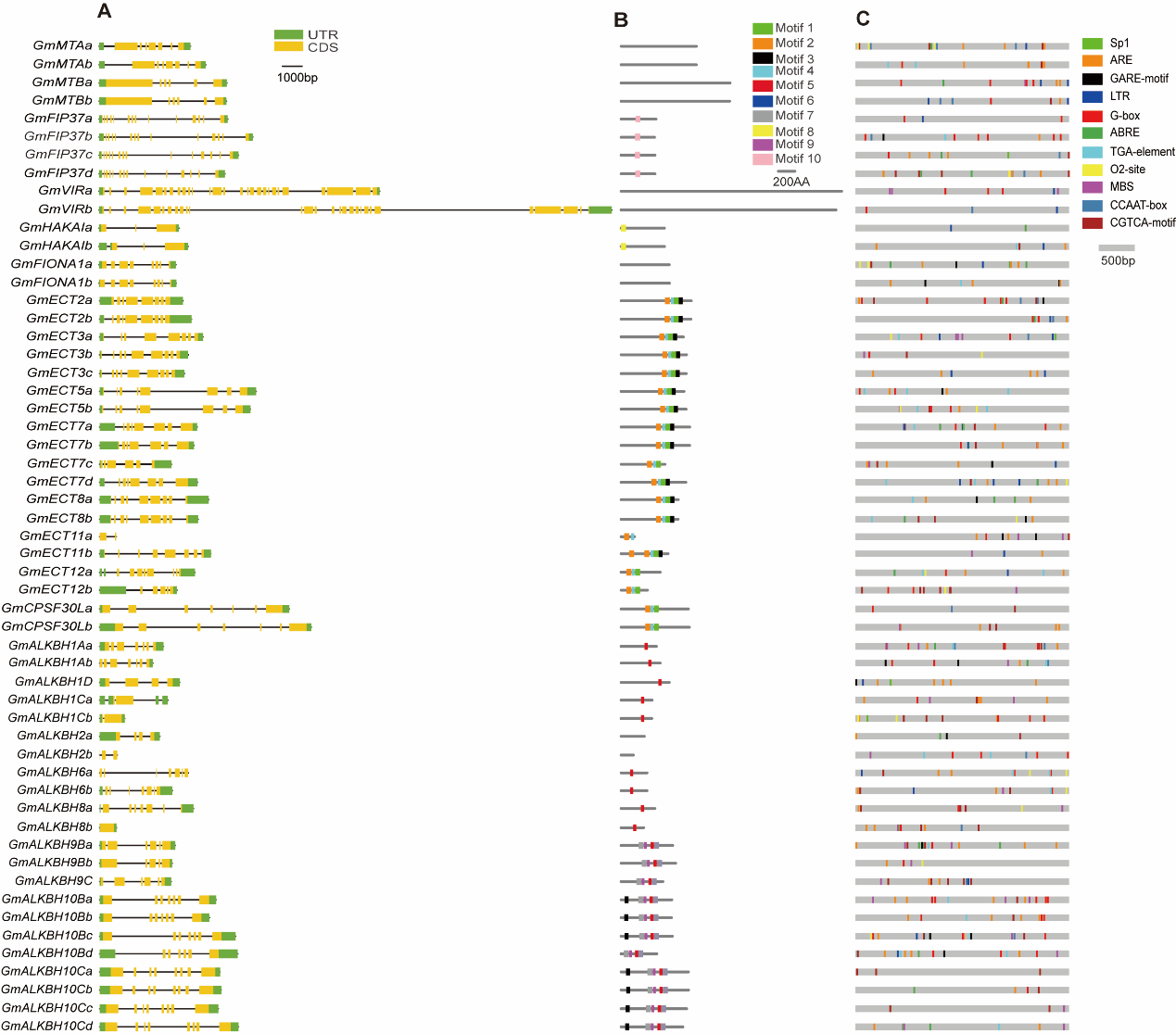


**Supplementary Fig. S1.** Structural and regulatory features of m^6^A-regulatory genes.

**A** Gene structure analysis showing intron-exon organization patterns of soybean m^6^A genes. **B** Distribution of conserved protein motifs in soybean m^6^A-regulatory proteins. Motif1-4: YTH domain, motif5-7: AlkB-like superfamily domain; motif8: KH_domain_RNA-binding. **C** Cis-regulatory element analysis in the promoter regions of soybean m^6^A genes.


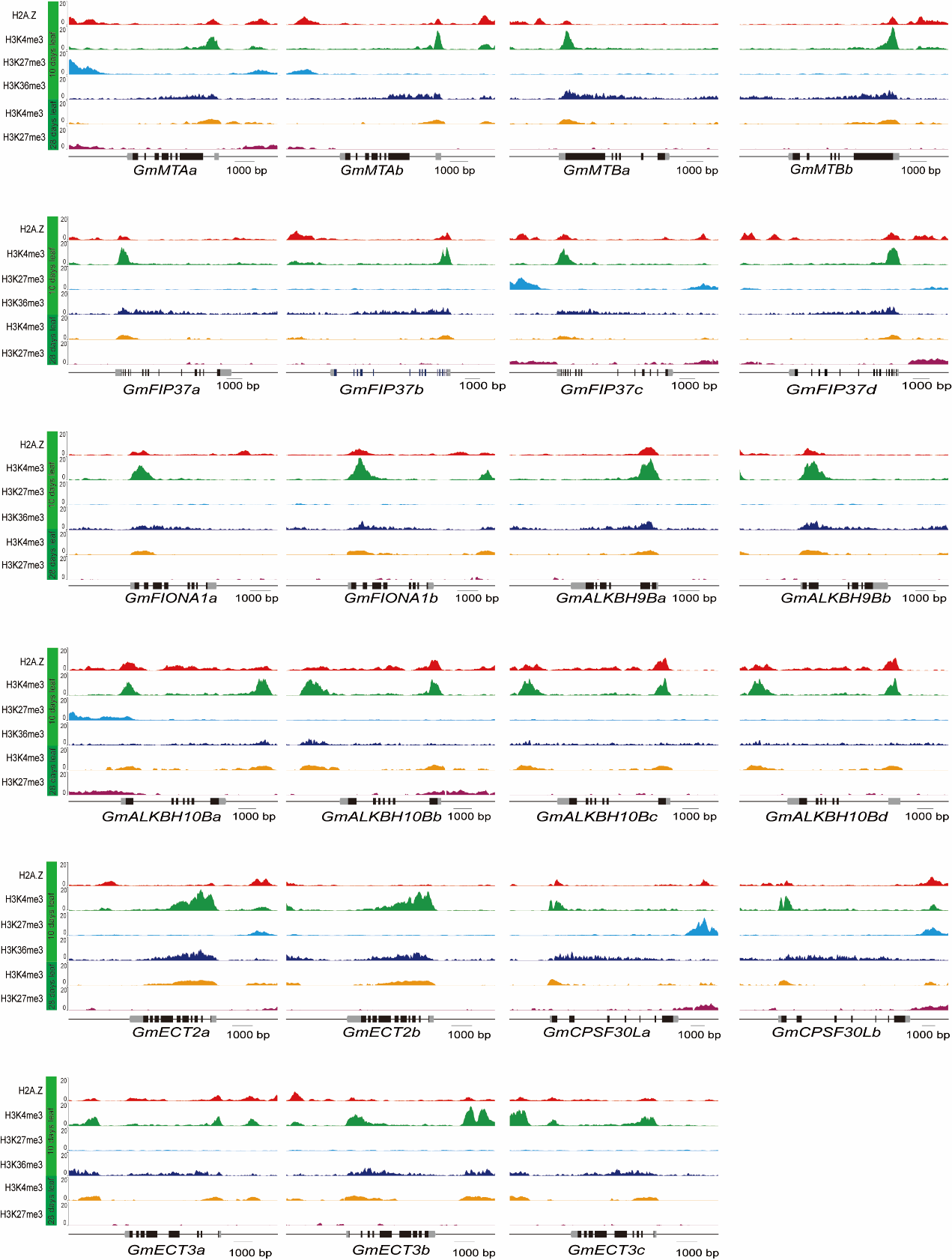


**Supplementary Fig. S2. Spatial distribution of histone modification enrichment patterns across selected soybean** m^6^A-regulatory **genes.**

The visualization illustrates the genomic localization of histone modification peaks associated with specific soybean loci. Numerical values within the figure indicate the range of enrichment values used for visualization scaling. **Supplementary**
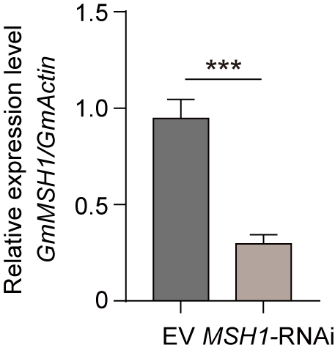
 **Fig.** **S3.** Relative transcript abundance of *GmMSH1* in EV and *msh1* mutant lines with 150mM NaCl treatment.

values are means ± s.d. of three biological replicates. Statistical significance was determined using two-tailed Student's *t*-tests and is denoted as follows: **p* < 0.05, ***p* < 0.01, ****p* < 0.001.


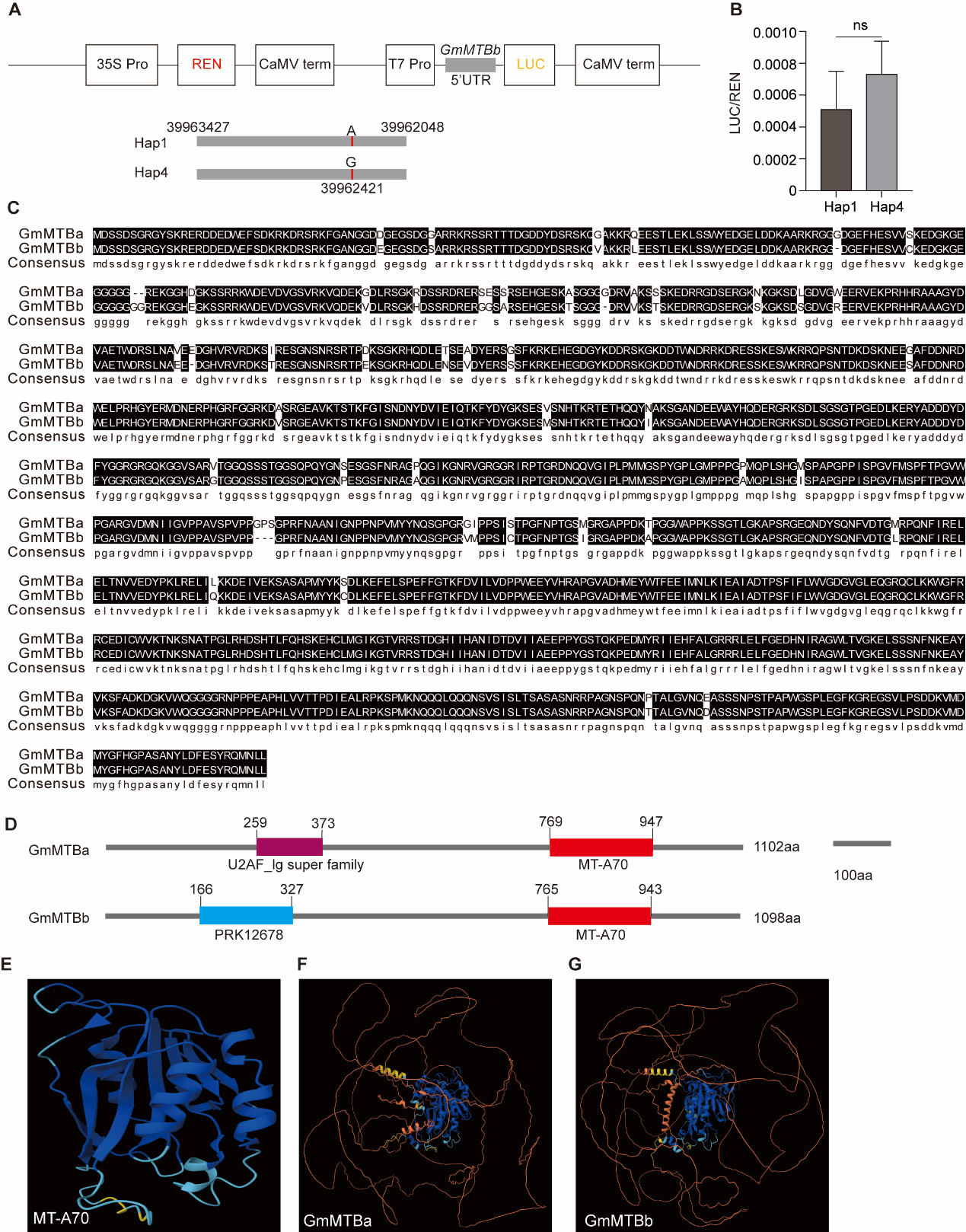


**Supplementary Fig. S4.** Sequence and structural variations of the soybean *GmMTBs* genes. **A** Schematic representation of diverse recombinant constructs applied in transient transfection assays on Nicotiana benthamiana leaf tissues, numbers in the figure correspond to the chromosomal localization of genes. **B** LUC/REN Activity Assay: *GmMTBb* 5'UTR Hap1 and Hap4 Constructs in N. benthamiana Leaves, Values are means ± s.d. of three biological replicates, statistical significance was determined using two-tailed Student's *t*-tests and is denoted as follow: ns *p>0.05*. **C** Amino acid sequence alignment: Black background indicates identical sequences. **D** Conserved domain prediction: The CD_Search tool was used for conserved domain analysis of amino acid sequences, Scale bar = 100 aa. **E-G** Three‐dimensional protein structures of MT-A70 domain, GmMTBa and GmMTBb generated with AlphaFold, E MT-A70 domain, F GmMTBa, G GmMTBb

**
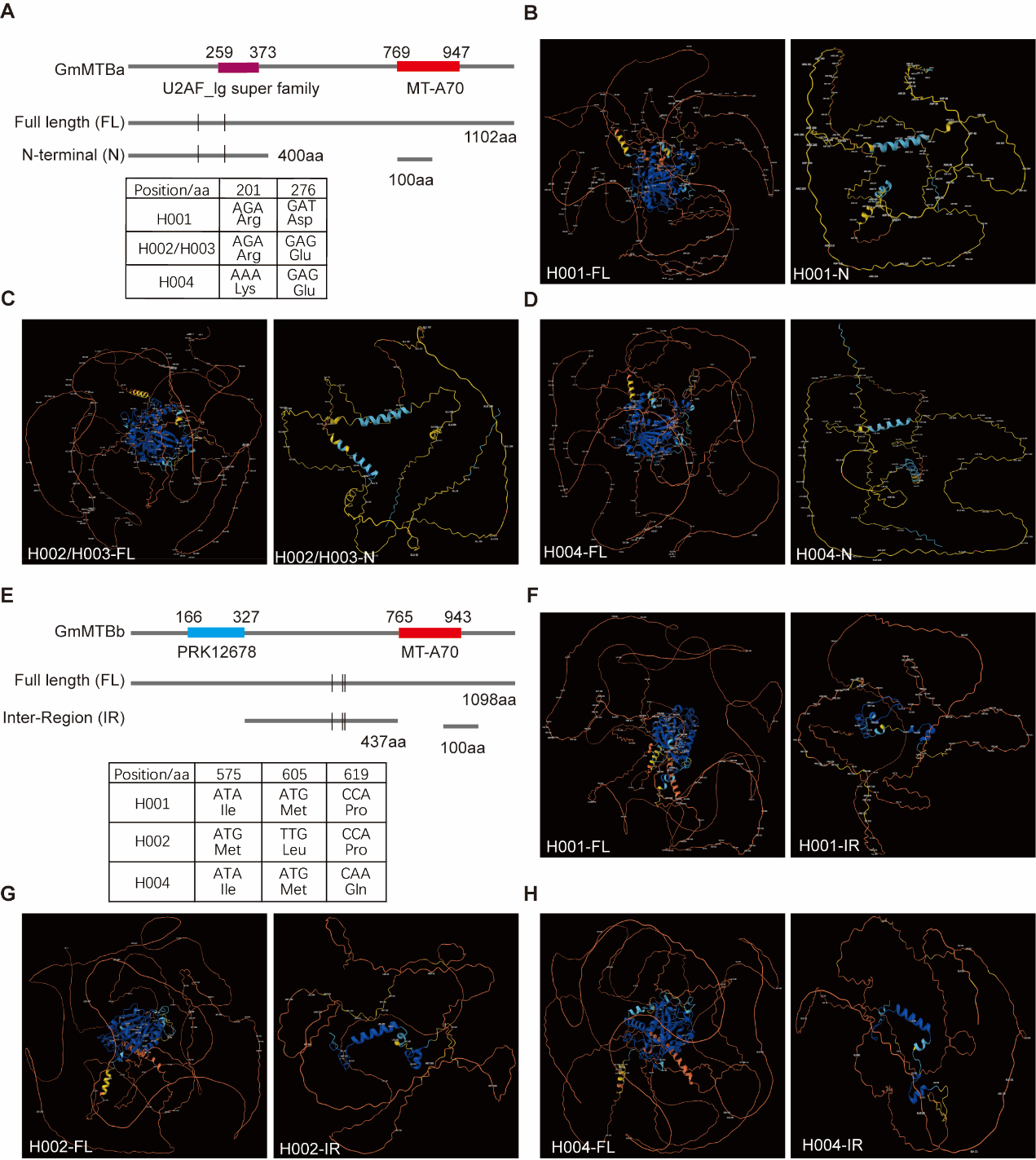
Supplementary Fig. S5.** Amino acid sequence and structural variations among different haplotypes of soybean *GmMTBs* genes. **A** Schematic diagram of sequence variations among different haplotypes of *GmMTBa*. Black vertical lines indicate variant sites, and the table on the right lists the positions and corresponding sequences of sequence variations; **B-D** Three-dimensional structures of different *GmMTBa* haplotypes predicted by AlphaFold, **B** *GmMTBa^H1^*, **C** *GmMTBa^H2/H3^*, **D** *GmMTBa^H4^*; **E** Schematic diagram of sequence variations among different haplotypes of *GmMTBb*. Black vertical lines indicate variant sites, and the table on the right lists the positions and corresponding sequences of sequence variations. **F-H** Three-dimensional structures of different *GmMTBb* haplotypes predicted by AlphaFold, **F** *GmMTBb^H1^*, **G** *GmMTBb^H^*^2^, **H** *GmMTBb^H4^*.
